# Development and Characterization of an Antimicrobial Polydopamine Coating for Conservation of Humpback Whales

**DOI:** 10.3389/fchem.2019.00618

**Published:** 2019-09-18

**Authors:** Ariana Tyo, Sonja Welch, Maureen Hennenfent, Pegah Kord Fooroshani, Bruce P. Lee, Rupak Rajachar

**Affiliations:** ^1^Engineered Biomaterials Lab, Department of Biomedical Engineering, Michigan Technological University, Houghton, MI, United States; ^2^Biomimetics Lab, Department of Biomedical Engineering, Michigan Technological University, Houghton, MI, United States

**Keywords:** polydopamine, antimicrobial, stainless steel, hydrogen peroxide, bacteria, coating

## Abstract

Migration patterns of humpback whales have been monitored using 316L stainless steel (SS) satellite telemetry tags. The potential for tissue infection and necrosis is increased if the bacteria, naturally a part of the diverse microbiome on the skin of humpback whales, can adhere to and colonize the surface of the tags. Polydopamine (pDA) has the potential to prevent the adhesion of one of the most prevalent bacterial strains on the surface of the skin of cetaceans (*Psychrobacter)* through the release of hydrogen peroxide. The release of hydrogen peroxide from the pDA coatings (40–100 μM) has the ability to induce a bacteriostatic response in *E. coli*, a commonly used bacteria strain in antimicrobial studies and potentially *P. cryohalolentis*, a common humpback associated bacteria. The H_2_O_2_ dose required to induce bacteriostatic conditions in *E. coli* is approximately 60 μM and in *P. cryohalolentis* is 100 μM. Bacterial adhesion on the surface of pDA coated SS coupons was measured first using *E. coli*. The coating successfully prevented adhesion of *E. coli* on the surface of SS coupons under certain conditions (60% reduction, *p* < 0.05) but the same was not seen with *P. cryohalolentis*. When coating conditions were altered (an increase in pH and temperature) the adhesion of *P. cryohalolentis* was reduced (80% reduction, *p* < 0.001). Overall, the pDA coatings have the capacity to generate varying amounts of hydrogen peroxide by altering the coating conditions and have the ability to reduce bacterial adhesion on the surface of satellite telemetry tags, and therefore the potential to increase tag functional service lifetime.

## Introduction

Understanding the migration patterns of large cetaceans, provides insight into global ocean temperatures, distribution of food sources, and population dynamics. Understanding where large cetaceans are traveling is the first step in most successful conservation efforts (Reeves et al., 2004; Lagerquist et al., 2008; Garrigue et al., 2010; Kennedy et al., 2014). The migration patterns of humpback whales (*Megaptera novaeangilae*), as well as other large cetaceans, have been monitored most recently using integrated satellite telemetry tags produced using 316L medical grade stainless steel (SS) (Garrigue et al., 2010; Kennedy et al., 2014). These tags have the potential to cause tissue damage or necrosis at the implant (tagging) site due to initial penetration into the skin-blubber layer as well as from other physical tag components such as retention elements (e.g., petals, barbs). During this process the tag comes into contact with a complex microbiome full of bacteria on the surface of healthy humpback whale skin (Bierlich et al., 2018). This microbiome, when on the surface of the skin, is harmless. However, during penetration there is potential for bacteria to migrate into the blubber tissue through stable adhesion and colonization on the surface of the telemetry tags. The most abundant bacterial genera found on the surface of humpback whales during tagging season are *Psychrobacter, Moraxellaceae, Tenacibaculum, and Flavobacterium* (Bierlich et al., 2018). The skin, similar to other mammals, contains multiple layers that can most generally be categorized into the epidermis, dermis, and hypodermis that together act as the first barrier to potential microbial insult with its thickness, relative smoothness, and sloughing acting to enhance the microbial barrier properties. The underlying blubber layer itself in composition and architecture is mainly a highly vascular adipose tissue containing a wide variety of fatty acids and a significant network (mesh) of collagen and elastic proteins to assist in maintenance of health and well-being in these mammals. The blubber is used to both store energy, mainly in the form of short-chain monounsaturated fatty acids (Borobia et al., 1995; Herman et al., 2009), and provide thermal insulation (Kvadsheim et al., 1996; Le, 2011). The blubber of humpback whales can be up to a foot thick and stores enough nutrients to manage one of the longest known migratory cycles among cetaceans (Herman et al., 2009).

Antibiotic coatings (e.g., aminoglycosides such as Gentamicin that disrupt bacterial protein synthesis and glycopeptides such as Vancomycin which inhibit peptidoglycan synthesis) (Hahn and Sarre, 1969; Watanakunakorn, 1984) have been successfully used on the surface of 316L SS implants to inhibit bacterial growth. Gentamicin in particular is the most commonly used antibiotic on cetacean telemetry tags, and has been able to successfully inhibit bacterial growth on tag surfaces. However, concerns regarding potential antimicrobial resistance (AMR) have made developing non-antibiotic based antimicrobial approaches of growing clinical importance. For this reason, the development and use of reactive oxygen species (ROS) based antimicrobial systems has become an area of interest (Vatansever et al., 2013; Meng et al., 2019). These species have been shown to have varying capacity to affect bacteria and mammalian cell type growth and survival on a dose-dependent basis. Examples of ROS species include superoxide anions ${\text{O}}_{2}^{-}$, peroxide ${\text{O}}_{2}^{-2}$, nitric oxide NO, and hydrogen peroxide H_2_O_2_. Polydopamine (pDA), a well-known marine adhesive (Lee et al., 2007b), derived from mussel foot proteins (Waite, 1999), has previously demonstrated antimicrobial properties against common land-based bacterial strains (Su et al., 2016). Polydopamine gets its adhesive and antimicrobial character from the presence of an abundance of catechols which have the ability to form strong bonds with both organic and inorganic surfaces, and when oxidized generate H_2_O_2_ (Lee et al., 2007b). Polydopamine has been used to create conformational adhesive coatings on the surface of organic and inorganic substrates. Common uses for these coatings include the detection of H_2_O_2_ (Li et al., 2016), acceleration of wound healing (Poh et al., 2010), and drug delivery (Liu et al., 2011). The coatings are typically applied using a dip coating procedure that takes up to 24 h (Liu et al., 2014; Ding et al., 2016; Su et al., 2016). The antimicrobial properties come from both the physical characteristics of the coating (i.e., roughness/thickness) and the generation of the ROS H_2_O_2_, that has previously demonstrated antimicrobial character and wound healing ability (Miyasaki et al., 1986; Linley et al., 2012). To balance these properties, it becomes important to distinguish between H_2_O_2_ dosing that is bactericidal (killing) and bacteriostatic (limiting growth via disruption of protein synthesis and reversible-irreversible adhesion). Polydopamine coatings will release H_2_O_2_ when oxidized (Salomaki et al., 2018) and can prevent the growth of bacteria, as well as potentially induce differentiation of adipose-derived stem cells (ASCs), to assist in the wound healing process. ASCs are common in human adipose tissue (Bunnell et al., 2008) and have been found in cetacean adipose tissue (Johnson et al., 2012). Low doses of ROS have been demonstrated to accelerate wound healing processes in mammals and, more specifically, doses of ROS (~50 μM) have resulted in differentiation of ASCs and accelerated healing. However, higher doses that are potentially bactericidal can result in poor or delayed wound healing outcomes (Lee et al., 2009; Johnson et al., 2012; Goudarzi et al., 2018). At one point H_2_O_2_–a commonly produced ROS that occurs naturally in the body at wound sites—was used as primary wound disinfectant, a practice that has become much less common with an increasing understanding of the dose dependent wound healing effects of H_2_O_2_ (Zhu et al., 2017).

This work aims to utilize the antimicrobial properties of pDA, through generation of H_2_O_2_, to create a coating on the surface of SS satellite telemetry tags that will induce a bacteriostatic condition that will ultimately aid local phagocytic cells in clearing bacteria as a result of a normal stable immune response while not sacrificing H_2_O_2_ and its ability to improve wound healing. The coating will prevent stable bacterial adhesion on the surface of the tags, allowing for more favorable microorganisms, or cells that assist in the wound healing response—such as prickle cells, to colonize the surface and decrease the chances for tissue infection at the tag implant site and potentially increase the effective tag service life (Parry, 1949).

## Materials and Methods

### Synthesis of Polydopamine (pDA) Surface Coating

**Figure 1:**
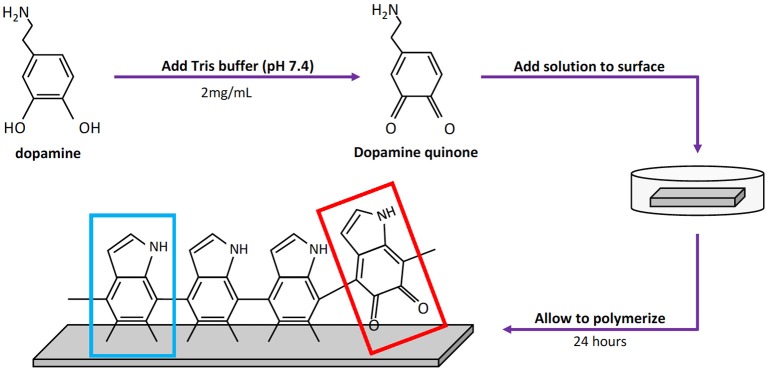
Process used to coat 316L Stainless Steel (SS) samples with poly-dopamine (pDA). To coat 316L SS samples, a pDA solution (2 mg/mL) was made using dopamine-HCl and a tris-HCl buffer (pH 7.4). After the tris-HCl buffer is placed onto the dopamine-HCl the dopamine will begin to oxidize immediately, creating a dopamine quinone. The solution is then placed on top of a 316L SS coupon and is allowed to polymerize for 24 h. Two forms of pDA will form: 5,6-dihydroxyindole (blue box) or 5,6-indolequinone (red box). The type of pDA found in the red box will not adhere to the surface of the metal.

Stainless steel coupons (10 mm × 20 mm) (McMaster-Carr, Elmhurst IL) were exposed to dopamine-HCl dissolved in a tris-HCl buffer (pH 7.4, Sigma Aldrich, St. Louis MO) at a concentration of 2 mg/mL and pDA was allowed to polymerize on the surface for 24 h at room temperature (Figure 1) (Terrill, 2015). After coating, coupons were gently shaken in PBS (pH 7.4) to remove non-adherent pDA from the surface. Coupons that were not used immediately after coating were air dried in a nitrogen-rich environment prior to use to avoid oxidation of the coating. Table 1 illustrates the coating condition modifications used throughout this study.

**Table 1:** pDA coating conditions.

**Condition Number**	**pH**	**Temperature, T (C)**	**Coating Time, CT (Hours)**
1	7.4	21	24
2	7.4	21	48
3	7.4	35	24
4	7.4	35	48
5	8.4	21	24
6	8.4	21	48
7	8.4	35	24
8	8.4	35	48

*H_2_O_2_ generation was evaluated from coupons coated under varying pH (7.4 and 8.4), temperature (21 and 35°C), and coating times (24 and 48 h). The highlighted row indicates the condition that generated the largest cumulative amount of H_2_O_2_ over 24 h*.

### pDA Surface Characterization

Standard [Roughness average (Ra) = 0.635 μm], brushed (Ra = 0.4 μm), and mirrored (Ra = 0.15 μm) finish 316L SS coupons (10 mm × 20 mm) were coated with pDA as previously described and were tested immediately after coating to prevent irreversible bonding of N_2_ to primary amine groups. Each surface was analyzed using X-ray Photon Spectroscopy (XPS) (PHI 5800, Physical Electronics, Chanhassen MN) with a binding energy of 1235.6 eV for atomic percent composition and thickness of the pDA coating.

**Figure 2:**
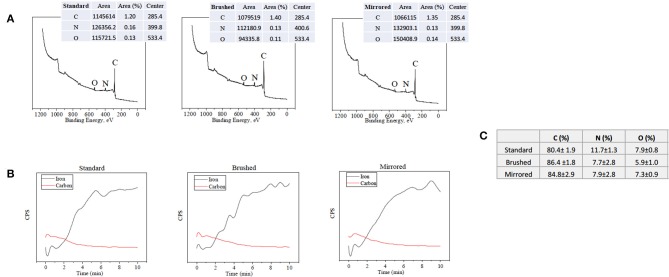
XPS data used for surface characterization. **(A)** Surface survey on standard, brushed, and mirrored surfaces used for atomic composition with labeled peaks and **(B)** corresponding depth profiles used for thickness measurements. **(C)** Table summarizing atomic composition of the coatings on each surface finish.

Atomic percent composition was calculated using survey spectral analysis (187.85 eV pass energy, 0.8 eV/step resolution, and 20 ms/step dwell time) at three random locations for each finish at a nominal diameter of 800 μm. The data was then analyzed in Origin® software (OriginLab Corporation, Northampton MA, USA) using a Gaussian-Lorenztian peak fit and quantified using the areas under the peaks. The peaks were matched to their element through the binding energies of C1s, N1s, and O1s, which are 284.8, 400, and 532 eV, respectively. An example of the peaks that were integrated on the various surface finishes can be seen in Figure 2A. Equation (1) below was then used to calculate atomic percent, where *A* is the area and *S* is the sensitivity factor of the element. The sensitivity factors used for Carbon, Nitrogen, and Oxygen are 0.296, 0.477, and 0.711, respectively (Briggs, 1981).

(1) $$\begin{array}{r} Atomic\;\%\;of\;element\;=\;\left(\frac{\frac{A}{S}}{\sum_{i=1}^{n}\frac{A_{i}}{S_{i}}}\right)\;x\;100\% \end{array}$$

Depth profiles were used to find the pDA coating thickness for each finish at three spots where main elements were tracked through a sputter and survey process at a sputter rate of 5.56 nm/min (2 kV Ar+) and sampling rate of 0.03 Hz. Carbon and iron were tracked and used to obtain thickness measurements. Carbon was used because of its high atomic percent in the pDA coating and low atomic percent in 316L SS. Iron ion (Fe^+2^) content was monitored to accurately assess where the coating ended and the underlying metal substrate began. Example of the curves produced from this process on all surface finishes are shown in Figure 2B. Thickness measurements were obtained by averaging the counts per second (CPS) value of the top and bottom plateaus, correlating that value to the time point, and multiplying by the sputter rate.

Contact angle measurements were conducted on all finishes of SS coupons coated under conditions 1 and 7 (see Table 1). Coupons were coated and allowed to air-dry for 6 h prior to testing. Coupons had a 5 μL drop of deionized water placed on top of the surface and images were taken to establish the contact angle of the droplet with the surface. Images were processed using ImageJ (NIH, v. 1.51). Degradation of the coating under aqueous conditions was also evaluated using phosphate buffered saline (PBS) pH 7.4 over 48 h. Coupons of each surface roughness were coated using conditions 1 and 7 (see Table 1) and were allowed to air-dry for 6 h. The mass of each SS coupon was measured before and after coating. The coupons were placed into PBS (pH 7.4) at 35°C for 48 h and the mass of the coupons was recorded at 48 h to determine the final mass, and compared to initial measurements.

### Dose-Dependent Growth Response of Marine Specific Bacteria to Hydrogen Peroxide

Dosing concentration of H_2_O_2_ required to induce a bacteriostatic response in *Psychrobacter Cryohalolentis* (ATCC BAA-1226) was determined by incubating cell suspensions (5 × 10^5^ CFU/well) with different initial doses of H_2_O_2_ (Calbiochem, San Diego CA) over a 48 h time period at 21°C (Bakermans et al., 2006). Solutions were allowed to incubate for various predetermined time points prior to measurements. Absorbance readings of optical density (OD) at 600 nm (OD_600_) were taken using UV-Vis turbidity measurements to determine cell concentration with respect to controls (Visible light UV-Vis, VWR Radnor PA).

### Evaluation of Hydrogen Peroxide Generation From pDA Coatings

Coupons were coated and the H_2_O_2_ generation was measured using a previously established protocol (Meng et al., 2019). Briefly, a ferrous oxidation-xylenol orange (FOX) colorimetric assay (Sigma Aldrich, St. Louis MO) was used to measure the H_2_O_2_ generation when the coupons were incubated at 21 and 35°C in a PBS solution at various time points. Concentrations of H_2_O_2_ in PBS solutions were determined by comparing to a control curve of known H_2_O_2_ in PBS. The total generation over 24 h was compared between all coating preparations (see Table 1).

### Bacterial Adhesion Response to pDA Coating

The most common bacteria on the surface of humpback whale skin, *Psychrobacter Cryohalolentis* (ATCC BAA-1226), and the most commonly used bacteria to evaluate antimicrobial effects, *Escherichia Coli* (ATCC 12435), were used to evaluate bacterial adhesion on the surface of coated 316L SS coupons with varying surface roughness. Coupons were coated using coating conditions 1 and 7 (see Table 1). These conditions were chosen as they have the lowest release—condition 1—and the highest release—condition 7—profiles. Coated coupons were incubated in a bacterial suspension (10^6^ CFU/mL) on a shaker table (120 rpm) for 24 h at 21°C and 5 h at 35°C, respectively. Samples were then fixed with 1% glutaraldehyde (Sigma Aldrich, St. Louis MO) for 12 h and then serially dehydrated in ethanol before imaging. Imaging was done using FE-SEM (Hitachi S4700, Schaumburg, IL) at 10 kV and 2,500x magnification. The number of bacteria cells in three separate areas on coated and uncoated SS coupons were manually counted to determine degree of bacterial adhesion (*N* = 3). Representative images used to evaluate bacterial adhesion on the surface of the SS coupons can be seen in Supplemental Figure 1.

### *In vitro* Biocompatibility

All biocompatibility tests were run on samples prepared using coating conditions 1 and 7 on brushed and standard surface finish coupons. L929 fibroblasts were seeded on top of SS coupons in a 6 well plate at a concentration of 10^5^ cells/well. Cells were allowed to adhere and grow for 24 h prior to staining with calcein-AM and ethidium bromide followed by fluorescence imaging. To assess proliferation, an MTT assay was performed after 24 h at an initial seeding density of 10^3^ cells/well in a 96 well plate. Standard cell counts were performed using ImageJ (NIH, v. 1.51) to validate results.

### Statistical Analyses

All statistical analyses unless otherwise specified were completed using a one-way ANOVA *t*-test. Statistical significance was set at a *p*-value of 0.05 (95% confidence interval) unless otherwise stated. Statistical analyses were performed using the data analysis toolkit in Microsoft® Excel for Mac Version 16.23. For comparing coating thickness, a two-sample *t*-test at a 95% confidence interval in Minitab was conducted to determine significance.

## Results

### pDA Surface Characterization

**Figure 3:**
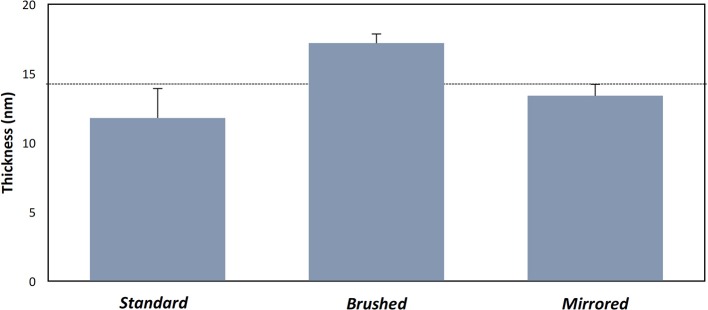
Thickness measurements of pDA coatings with varying substrate roughness. Thickness measurements of the coating on various surface finishes from XPS measurements. The dotted line represents the mean thickness for all measurements. There were no significant differences found between samples.

Polydopamine coatings were successfully created and characterized through atomic percent composition and thickness measurements. The intensity of the peaks corresponding to C1, O1, and N1 were used to determine atomic percent of each element (Figure 2A). The atomic percent did not significantly change between surface finishes (Figure 2C). The average coating thickness determined by the quantity of Fe^+2^ ions present from depth profile measurements was 11.8 ± 2.11 nm, 17.2 ± 0.66 nm, and 13.4 ± 0.82 nm on standard, brushed, and mirrored finishes, respectively, with a mean thickness for all coated samples of 14.1 ± 2.64 nm (Figure 3). As expected, there was no significant difference found between the coating thicknesses for each surface finish.

Contact angle measurements indicated a hydrophilic surface (θ ≈ 20°, where θ represents the surface contact angle; θ = 0° for complete wetting and θ = 180° for non-wetting) on all pDA coated surfaces (data not shown). The pDA coating did not significantly alter the hydrophilicity of the uncoated SS coupons. Additionally, all coating formulations, regardless of the initial coating conditions (Condition 1 and Condition 7), did not degrade when incubated for 48 h in PBS at pH = 7.4 as determined by the initial and final mass differences.

### Hydrogen Peroxide Generation and Bacterial Growth Response

**Figure 4:**
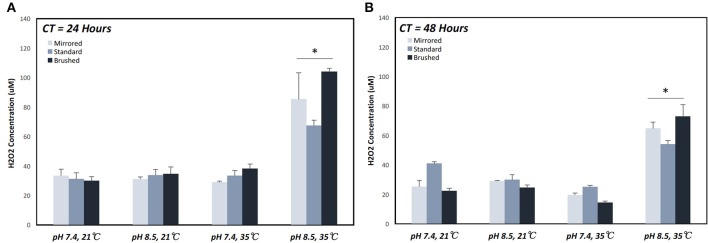
Cumulative hydrogen peroxide release of pDA coatings created under various conditions over a 24 h period. Hydrogen peroxide release measured over a 24 h period on coupons coated for **(A)** 24 and **(B)** 48 h. The highest release was seen on coupons coated with pDA using a pH 8.5 solution at 35°C for 24 h. When coated for 48 h there was a smaller increase in release when increasing temperature for pH 8.5 and a slight decrease in release for pH 7.4 with increasing temperature. *A significant difference from all other coating conditions within the surface finish (*p* < 0.05). More broken-down results with all significant differences for individual surface finishes can be found in Supplemental Figure 2. CT, coating time.

The generation of H_2_O_2_ was measured from coupons under various coating conditions (Table 1). The maximum H_2_O_2_ production over a 24 h period (67.44 μM ± 6.4 μM) was achieved under coating condition 7 (pH = 8.4, T = 35°C, and CT = 24 h) (Figure 4A). At CT equal to 24 h, pH alone did not make a significant difference in H_2_O_2_ release behavior for all surface finishes whereas an increase in both pH and temperature resulted in approximately a 2-fold increase on all surface finishes (Supplemental Figure 2). This trend was also observed with a CT equal to 48 h. However, with a CT equal to 48 h and at lower temperatures an increase in pH effectively reduced H_2_O_2_ production on the standard surface finish (40.1 μM ± 2.0 μM [pH 7.4] vs. 29.88 μM ± 5.89 μM [pH 8.4]), while both mirrored and brushed finishes showed no significant change in release of H_2_O_2_ (Figure 4B).

**Figure 5:**
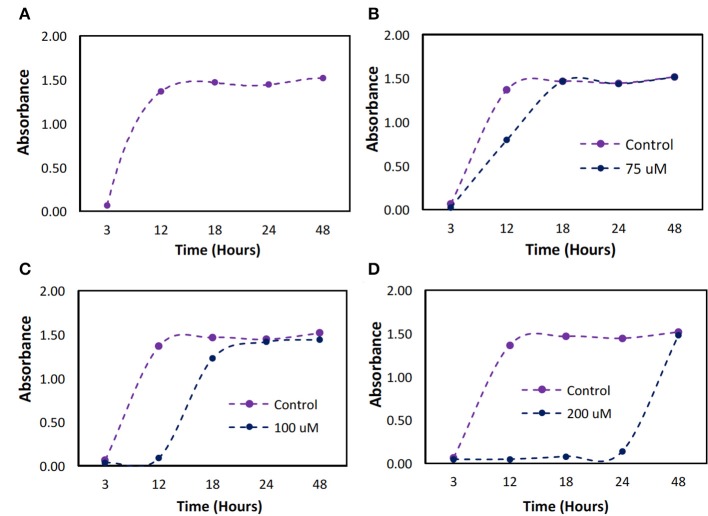
Bacterial growth over a 48 h period with different initial doses of hydrogen peroxide. *P. cryohalolentis* bacteria growth was measured using absorbance readings at 600 nm with different initial doses of H_2_O_2_. **(A)** The control was used to determine typical bacterial growth. **(B)** Growth was first slowed at a dose of 75 μM. **(C)** At 100 μM dosing, bacteriostatic conditions were achieved. **(D)** At 200 μM bacteriostatic conditions were maintained for 24 h.

The minimum dose required to establish bacteriostatic conditions for *P. cryohalolentis* was previously unknown. The bacteria, when untreated, grew to full confluence (turbidity = 1.5) within 12 h (Figure 5A). We found a one-time initial dose of 100 μM was required to maintain bacteriostatic conditions for 12 h (Figure 5C). A bacteriostatic condition could be maintained for 24 h with the administration of 200 μM H_2_O_2_ (Figure 5D) however this dose has been shown to be toxic to ASCs.

### Bacterial Adhesion Response to pDA Coating

**Figure 6:**
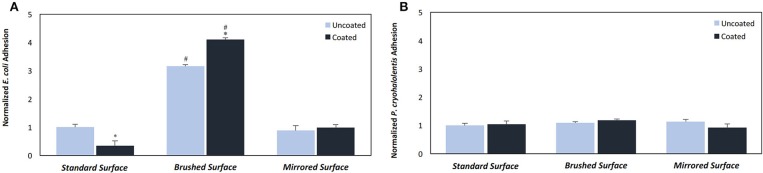
Bacterial adhesion on coated and uncoated SS coupons for **(A)** *E. coli* and **(B)** *P. cryohalolentis*. Coatings were created in pH 7.4 and 21°C with a CT = 24 h. Normalized bacterial adhesion of **(A)** *E. coli* to the surface of 316L SS coupons after incubating in a bacterial solution for 5 h and **(B)** *P. cryohalolentis* to the surface of 316L SS coupons after incubating in a bacterial solution for 24 h. ^#^A significant difference from the respective standard surface finish (*p* < 0.05); *A significant difference from the respective uncoated surface (*p* < 0.05) using a standard one-way ANOVA *t*-test.

**Figure 7:**
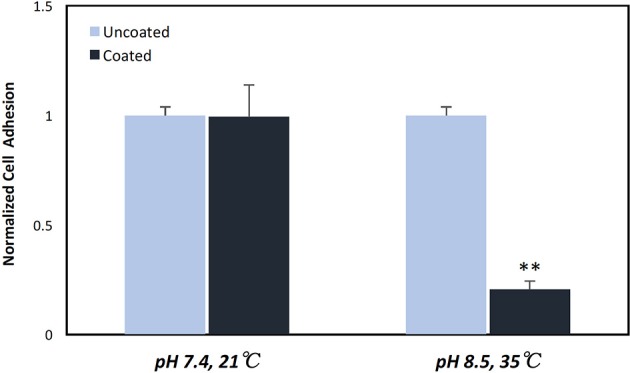
*P. cryohalolentis* bacterial adhesion on coated and uncoated standard finish SS coupons. Coatings were created in pH 7.4 at 21°C and pH 8.5 at 35°C. Normalized bacterial adhesion of *P. cryohalolentis* to the surface of 316L SS coupons after 24 h showed no significant change with the pH 7.4 solution, however, there was a significant reduction in bacterial adhesion on the surface of SS coupons with the pH 8.5 coating. **A significant difference from both the uncoated SS and the other coating condition (*p* < 0.001) using a standard one-way ANOVA *t*-test.

Having established that pDA coatings created under coating condition 1 (pH = 7.4, T = 21°C, CT = 24 h) were relatively uniform in thickness and composition (i.e., no statistically significant differences), *E. coli* displayed a greater adherence sensitivity to differences in coatings on substrates of varying roughness when compared to *P. cryohalolentis* (Figures 6A,B). For *E. coli* the number of adherent bacteria was significantly reduced (*p* < 0.05) on the standard surface 316L SS with the addition of the pDA coating and increased significantly (*p* < 0.05) on brushed SS coupons—both coated and uncoated. The adhesion was further increased over the uncoated brushed SS with the addition of the pDA coating onto the surface. The mirrored surface showed no significant difference in *E. coli* adhesion from the standard surface with or without a pDA coating (Figure 6A). *P. cryohalolentis* adherence was relatively insensitive to changing surface roughness or with the addition of a pDA coating under coating condition 1 (Figure 6B). Importantly, under coating condition 7 (pH = 8.5, T = 35°C, CT = 24 h) *P. cryohalolentis* adhesion was significantly decreased when compared to the uncoated control and coating condition 1 (*p* < 0.001) (Figure 7).

### *In vitro* Biocompatibility

**Figure 8:**
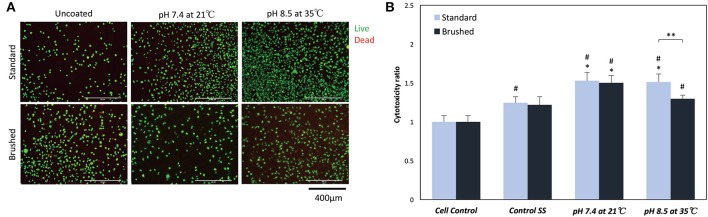
**(A)** Live/Dead and **(B)** MTT cell biocompatibility assays. **(A)** Representative images on the surface of coated and uncoated 316L SS coupons. There was no significant change in the number or morphology of fibroblasts numbers on the surface of SS coupons. **(B)** MTT proliferation assay results showed a significant increase in proliferation when fibroblasts were exposed to low doses of hydrogen peroxide. *A significant difference from respective SS controls (*p* < 0.05); ^#^A significant difference from cell controls (*p* < 0.05); **A significant difference between SS finishes at an increased pH and temperature (*p* < 0.05).

Live/dead staining indicated no significant change in cell morphology or in the presence of dead (red) cells for all sample groups (Figure 8A). The MTT assay showed significant differences in proliferation behavior with the addition of pDA coatings (Figure 8B). Fibroblasts seeded on uncoated standard SS finish coupons had increased proliferation over cell controls, while uncoated brushed SS coupons did not exhibit increased proliferation. When a pDA coating was added to the substrate surfaces, the proliferation significantly increased over cell controls for both coating conditions 1 (pH = 7.4, T = 21°C, CT = 24 h) and 7 (pH = 8.5, T = 35°C, CT = 24 h) and surface finishes. However, under coating condition 7 there was significantly more cell proliferation on the standard surface when compared to the brushed surface (*p* < 0.05) (Figure 8B).

## Discussion

Stainless steel telemetry tags are a useful tool to conservation scientists as they help monitor the migration patterns of large cetaceans. The tags, however, need to pass through a layer of skin that has a diverse microbiome which can potentially lead to infection at the implant site. Reducing susceptibility to infection is one of the methods for improving long-term biocompatibility and ultimately increasing the service life of tags by creating a microenvironment more suitable for wound healing. With the growing interest in developing robust non-antibiotic approaches, polydopamine surface coatings have the potential to modulate stable marine bacterial colonization to an implant surface with the controlled release of the ROS H_2_O_2_. Furthermore, since H_2_O_2_ is also a known effector of stable wound healing, controlled delivery in a target range could not only facilitate a bacteriostatic behavior conducive to the inherent immune modulation of infection but it could also be used to promote stable wound healing via the stimulation of resident effector cells that include adipose derived stem cells (ASCs).

The pDA surface coatings in this work were established to be conformational and stable in solution on varying surface finishes of 316L stainless steel (SS). The composition and thickness of the coatings remained the same for all finishes as well. The coatings did not exhibit any significant differences in chemical composition when the substrate roughness was altered (Figure 2C). This relative substrate uniformity and stability is expected as the substrate roughness is not a contributing factor to characteristic changes in pDA coatings (Ding et al., 2016). Previous studies have established, using a solution with a pH of 8.5, a pDA coating with a thickness of 50 nm can be created (Lee et al., 2007a). In this study, the average coating thickness obtained using a solution with a lower pH of 7.4 ranged from approximated 11–17 nm (Figure 3). In this work the pH used to create the coating, and therefore the corresponding thickness of the coating, did not significantly affect the coatings release behavior (Figure 4 and Supplemental Figure 2). Thus, the pDA coatings in the current study have a tailored H_2_O_2_ release profile that can be easily adjusted by altering the pH, time, and temperature during the coating process and that is relatively independent of coating thickness. This release profile can be adjusted to induce bacteriostatic responses from *E. coli* and *P. cryohalolentis*. Coating condition 1 (pH = 7.4, T = 21°C, and CT = 24 h) was able to induce a bacteriostatic response in *E. coli* but not *P. cryohalolentis* due to a lower required H_2_O_2_ threshold to induce a bacteriostatic response for *E. coli* shown in other studies (Figure 6A) (Juven and Pierson, 1996). There was no bacteriostatic response for *P. cryohalolentis* using coating condition 1 as this release profile was below the established threshold required to induce a bacteriostatic response in *P. cryohalolentis* bacteria (Supplemental Figure 3). Coating condition 7 (pH = 8.5, T = 35°C, CT = 24 h), which has a significantly higher cumulative release when compared to condition 1 (~30 μM vs. ~100 μM) and met the threshold previously established, was able to induce a bacteriostatic response and decreased the adhesion of *P. cryohalolentis* bacteria to the surface of standard SS (Figure 7). The threshold for adipose cell death of humpback whales is currently unknown so the ability to tailor the release character of H_2_O_2_ to this threshold will be of importance in determining the balanced release profile needed to promote both an antimicrobial-bacteriostatic effect and stable wound healing.

The variation in release of H_2_O_2_ can be attributed to coating conditions which can alter the chemical arrangement (catechol exposure) of the coatings—therefore altering the H_2_O_2_ release profiles (Salomaki et al., 2018). Both the temperature and the pH of the coating solution affected release profiles. Previous experiments have concluded that increasing the incubation temperature will increase the rate at which H_2_O_2_ is generated in a solution with a constant pH due to an increased oxidation state (Wang et al., 2016). In addition, it has been determined that a pH between 8 and 9 will produce a greater amount of H_2_O_2_ than a pH between 6 and 7 on non-metal substrates (Torres et al., 2014). However, there is a lack of literature on the alteration of coating conditions and what effect this may have on H_2_O_2_ release. It is well-known that coating conditions alter the chemical makeup of the coatings, as warmer and more basic conditions allow for more catechol side groups to be available for interaction. It is not well-studied whether or not these changes contributed to an increased H_2_O_2_ release on the surface of metal substrates.

Bactericidal and bacteriostatic conditions can be induced using H_2_O_2_ for both *E. coli* (Miyasaki et al., 1986) and *P. cryohalolentis* (Figure 5). Adherence of *E. coli* was decreased with the addition of the pDA surface coating on the standard surface within the incubation time (5 h), this may be explained by the significant increase (*p* < 0.05) in H_2_O_2_ generation on the standard surface early in the incubation period over both the brushed and mirrored surfaces (Supplemental Figure 4). A one-time dose of 100 μM can induce bacteriostatic conditions for 12 h in *P. cryohalolentis* however a low, sustained dose can induce a bacteriostatic response in solution for up to 16 h (Supplemental Figure 5). While bacteria will adhere to the surface of coated SS coupons initially, with a prolonged release of H_2_O_2_ the bacteria will detach from the SS after 24 h (Supplemental Figure 5). At a concentration of 100 μM ASCs have a lower survival rate if they are not already confluent in culture (Lee et al., 2009). Due to this threshold in both ASCs and *P. cryohalolentis*, it is possible a pDA coating producing 100 μM of H_2_O_2_ over 24 h would have the ability to prevent the colonization of bacteria on the surface of the tag as well as assist in advanced wound healing within the tissue.

## Conclusion

Polydopamine coated on 316L stainless steel was able to reduce the adhesion of *E. coli* and *P. cryohalolentis*. The coating is simple to make and can be easily tailored to alter the amount of H_2_O_2_ generated through adjustments in the coating environment. Creating a coating that can assist in the differentiation of ASCs and prevent stable bacterial adhesion and colonization to the surface of satellite telemetry tags can prove to be useful in conservation practices. Future work will focus on further evaluation of *P. cryohalolentis* behavior on coatings created in different conditions with varying substrate roughness, the adhesion of the second most common bacteria species found on the surface of humpback whales (*Tenacibaculum*) to the surface of coated and uncoated 316L SS coupons, and the differentiation of marine mammal ASCs when exposed to low doses of H_2_O_2_ as well as our pDA surface coatings.

## Data Availability

All datasets generated for this study are included in the manuscript/Supplementary Files.

## Author Contributions

AT, BL, and RR designed and directed the project. AT and PK designed and completed all the bacterial experiments. SW designed and performed the characterization experiments. MH and PK performed and analyzed FOX assays. AT, SW, and MH formatted and analyzed the data. AT took lead on assembling the manuscript. All authors contributed to the final version of the manuscript. BL and RR supervised the project and were in charge of overall direction of the project.

### Conflict of Interest Statement

The authors declare that the research was conducted in the absence of any commercial or financial relationships that could be construed as a potential conflict of interest.
