# Supplementary figures and images for: Development and Characterization of an Antimicrobial Polydopamine Coating for Conservation of Humpback Whales
Source: Front Chem. 2019 Sep 18;7:618. doi: 10.3389/fchem.2019.00618 (PMC6759777; doi:10.3389/fchem.2019.00618)

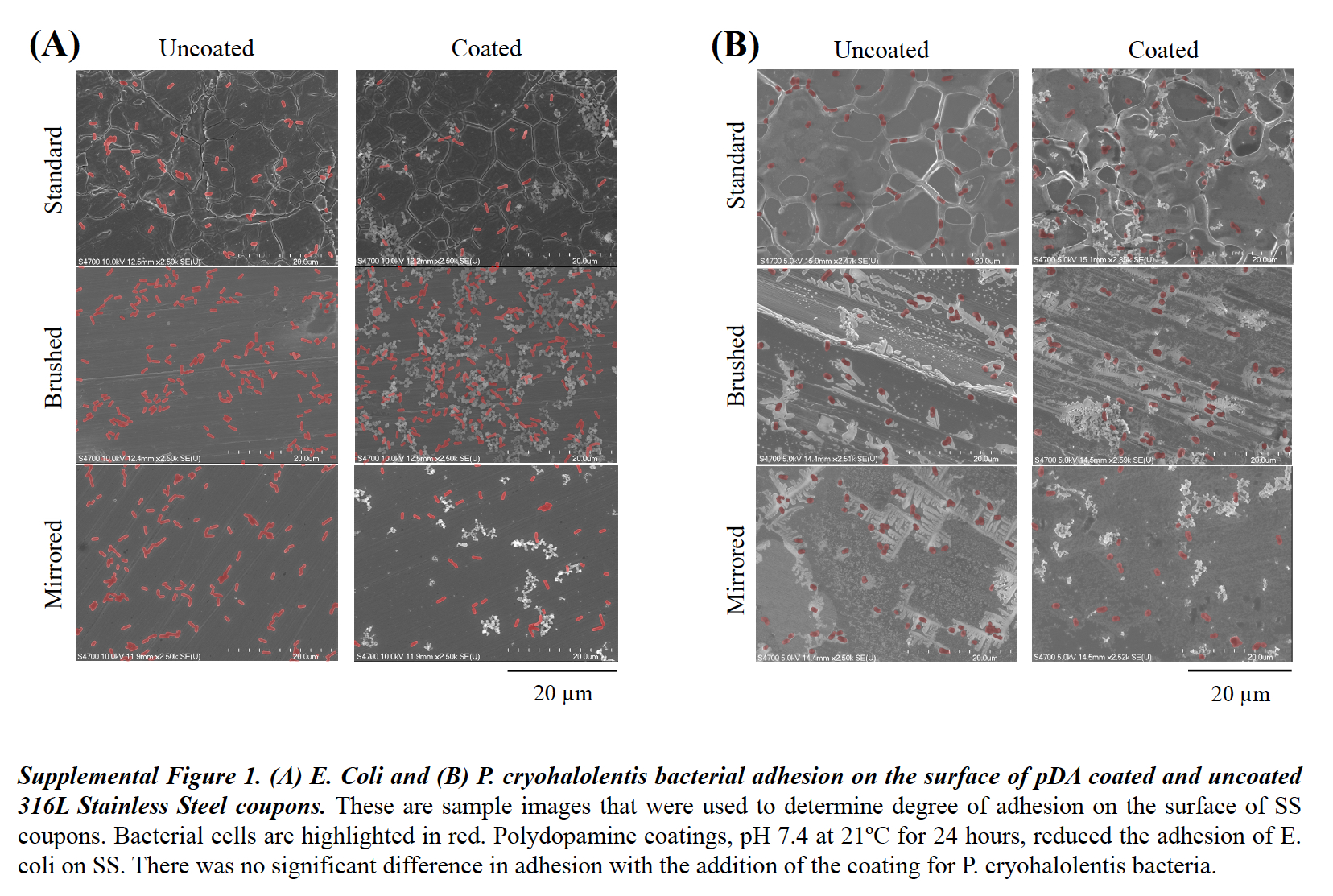

Supplement: Supplementary file 1 [file Image_1.TIFF]

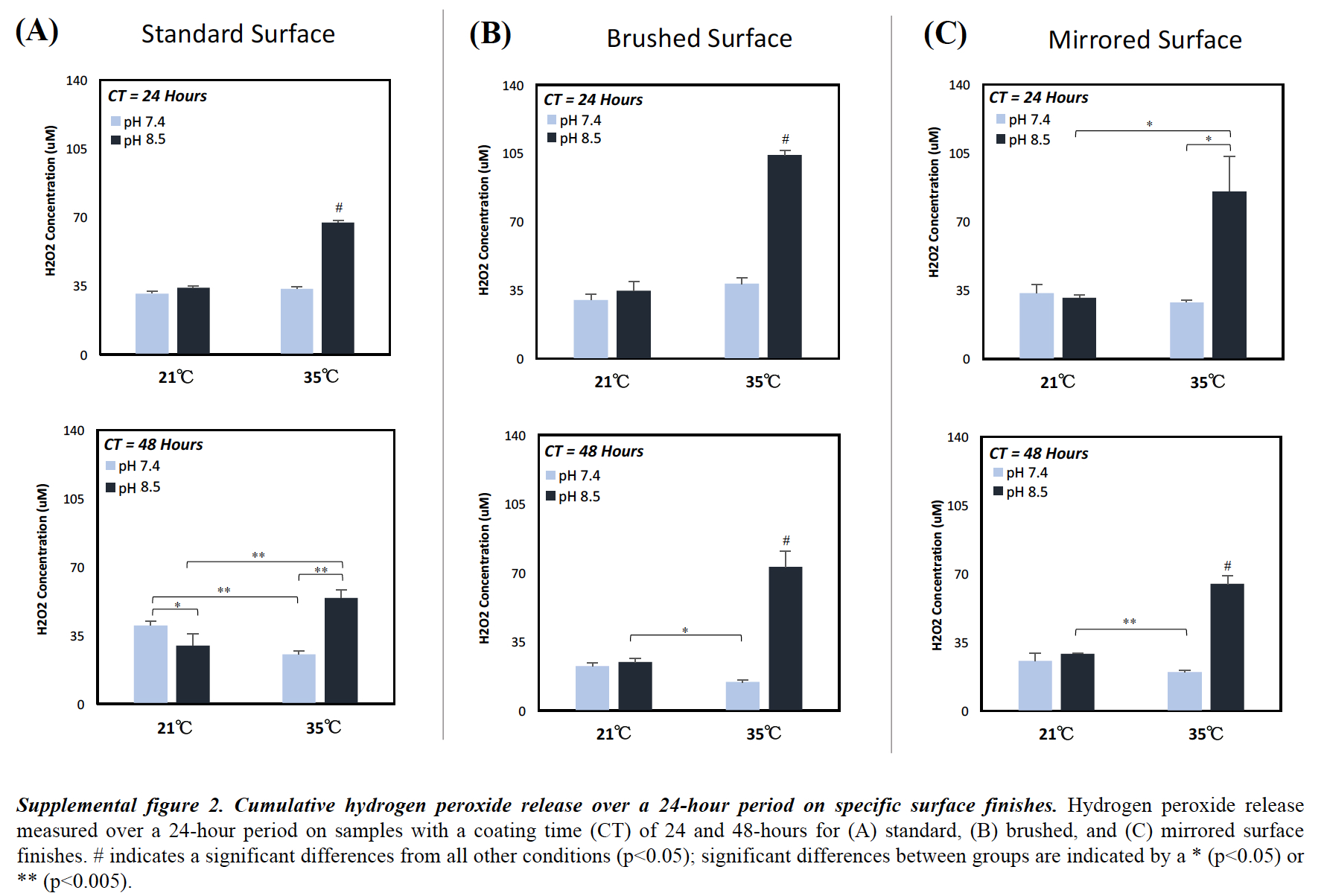

Supplement: Supplementary file 2 [file Image_2.TIFF]

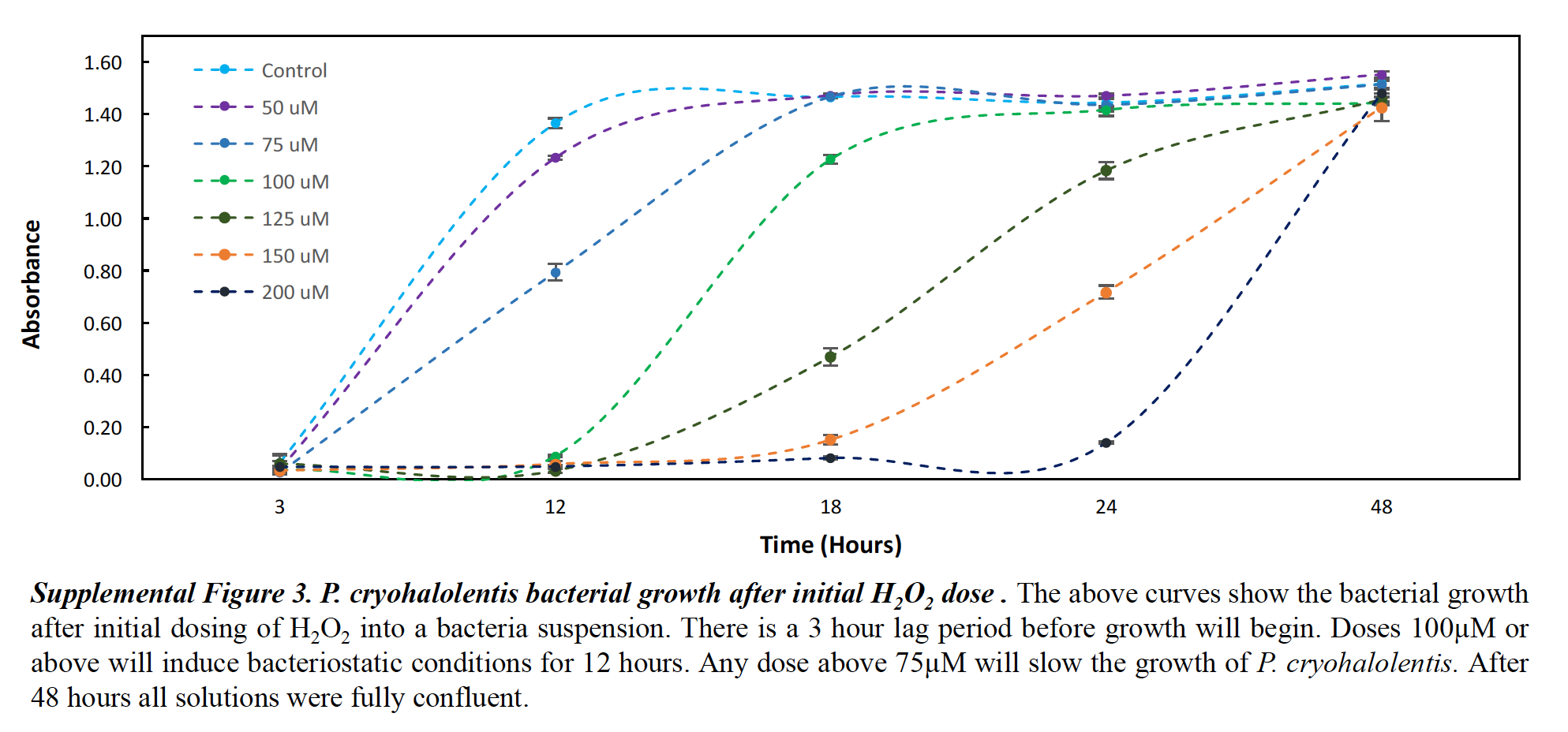

Supplement: Supplementary file 3 [file Image_3.TIFF]

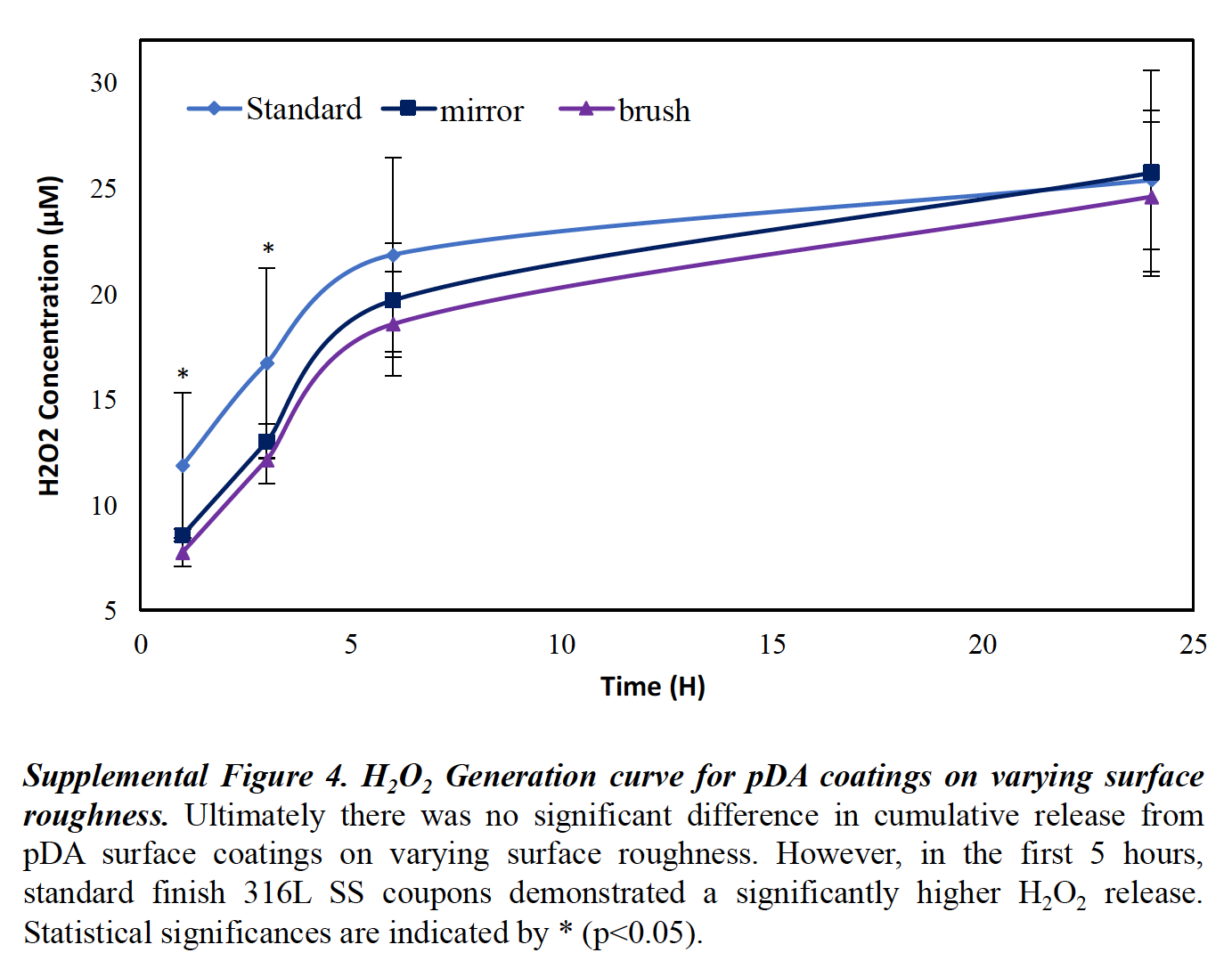

Supplement: Supplementary file 4 [file Image_4.TIFF]

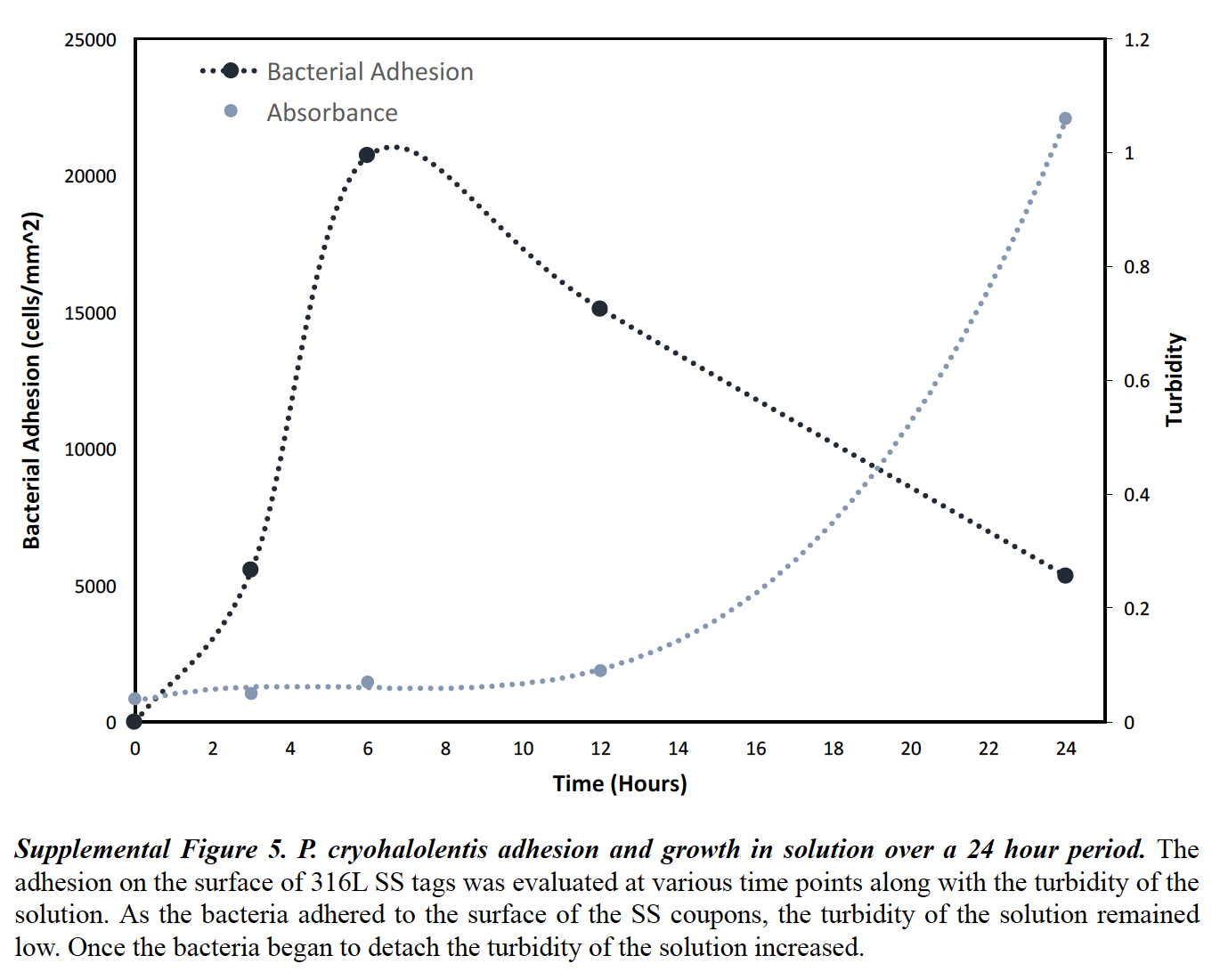

Supplement: Supplementary file 5 [file Image_5.TIFF]
